# Lymphoma Heterogeneity Unraveled by Single-Cell Transcriptomics

**DOI:** 10.3389/fimmu.2021.597651

**Published:** 2021-02-26

**Authors:** Loic Ysebaert, Anne Quillet-Mary, Marie Tosolini, Frederic Pont, Camille Laurent, Jean-Jacques Fournié

**Affiliations:** ^1^Centre de Recherches en Cancérologie de Toulouse, INSERM UMR1037, Toulouse, France; ^2^Toulouse University, Toulouse, France; ^3^ERL 5294 CNRS, Toulouse, France; ^4^Institut Universitaire du Cancer-Oncopole, Toulouse, France; ^5^Laboratoire d’Excellence ‘TOUCAN’, Toulouse, France; ^6^Institut Carnot Lymphome CALYM, Lyon, France; ^7^Service d’Hématologie, CHU Toulouse, Toulouse, France; ^8^Laboratoire d’anatomo-pathologie, CHU Toulouse, Toulouse, France

**Keywords:** single-cell RNA sequencing (scRNA-seq), lymphoma, microenvironment, bioinformatics analysis, cell of origin (COO)

## Abstract

High-definition transcriptomic studies through single-cell RNA sequencing (scRNA-Seq) have revealed the heterogeneity and functionality of the various microenvironments across numerous solid tumors. Those pioneer studies have highlighted different cellular signatures correlated with clinical response to immune checkpoint inhibitors. scRNA-Seq offers also a unique opportunity to unravel the intimate heterogeneity of the ecosystems across different lymphoma entities. In this review, we will first cover the basics and future developments of the technology, and we will discuss its input in the field of translational lymphoma research, from determination of cell-of-origin and functional diversity, to monitoring of anti-cancer targeted drugs response and toxicities, and how new improvements in both data collection and interpretation will further foster precision medicine in the upcoming years.

## Introduction

Like any other cancer, lymphomas are heterogeneous diseases exhibiting molecular aberrations at multiple levels. Adding difficulties to the understanding of lymphomagenesis, interactions with bystander stromal and immune cells in specific, highly organized microenvironments (ME) dictate tumor cell behavior. Since 2000, bulk transcriptomic and mutational profiles have been extensively characterized from pooled, heterogeneous mixtures of both cancer and ME cells, leading to the first classifications based on *cell of origin*. In diffuse large B-cell lymphomas (DLBCL, the most common lymphoma subtype), prognosis after chemo-immunotherapy differs according to germinal center or non-germinal center transcriptomic signatures. In follicular lymphoma (FL, second most frequent entity), prognosis is better predicted by the type of ME cells (T cells *versus* macrophages), rending interpretation of data dependent of the type and abundance of cells in a single biopsy specimen.

Single-cell RNA-sequencing (scRNA-Seq), by shedding light on gene expression levels across thousands of cells mixed into a patient’s biopsy without sorting, has revolutionized our understanding of normal human tissues’ anatomy, ontogeny, and diseases (1–3). Thanks to this technology, dissecting tumor heterogeneity is now increasingly an achievable goal in cancer care (4, 5). Indeed, development of resistance to most recent targeted agents originates both from tumor and ME transcriptomic variability, the latter directly influencing lymphoma phenotypic heterogeneity. According to Darwinian laws, evolution selects the fittest phenotype, not genotype. Several studies have confirmed that genetic variations are observed in distinct ecosystems within the same tumor, and that spatial distribution of cellular subsets with specified transcriptomic signatures correlates to clinical outcome (6, 7). Lymphomas are a group of lymphoid tumors with widespread body dissemination (though not considered *metastases*), invading blood and lymph vessels. They are therefore asking daily the question of how to interpret a selective biopsy of tumor sample in the light of cancer heterogeneity.

Other reviews have provided excellent historical perspectives on the emergence and development of increasingly cost and time saving scRNA-Seq protocols, and how the commercial platforms now allow dissemination of knowledge in many Centers treating patients (4, 6, 8–10). After going through technological considerations about advantages/disadvantages of scRNA-Seq as compared to other techniques, our goals are to inform readers about latest insights in basic and translational lymphoma research about:

-*Cell of origin* of tumor cells
-Functional and phenotypic heterogeneity of lymphomas
-Inputs in clinical research: monitoring the response to therapy, and defining markers of early progression/toxicity (with an emphasis on the latest anti-lymphoma armamentarium: cellular therapies (CAR T-cells), and immune checkpoint blockers)

## Bulk RNA Analyses: What Have We Learned About Intra- and Extra-Tumor Heterogeneity in Lymphoma Over 20 Years?

Malignant lymphomas mirror the complexity of immune system by many aspects. Since the advent of whole transcriptome profiling by Affymetrix-based microarrays, transcriptomics of tumor samples has enabled the identification of various molecular subsets of cancer cells, as originally the differential profiles of germinal center (GC)-like and activated B cell type (ABC) diffuse large B-cell lymphoma (DLBCL) defining cell (11). This has led to a better characterization of entities (>90 in the WHO2018 classification).

The genuine technology consisted in capturing each mRNA from a cellular lysate thanks to arrays of thousands oligonucleotide probes, each specific for a defined gene, and quantifying the captured mRNA by fluorescence signals (11). This allowed to quantify quite precisely the expression level of each gene taken individually, an information which once paralleled across the ~20,000 human genes, provided a global view of most cellular hallmarks of the cell types within the analyzed sample. Further direct sequencing of the mRNAs (RNA-seq) from bulk cell samples improved the sensitivity and precision of transcriptomes over the former microarrays, but did not revolutionize significantly the quality of the results: the microarray and RNA-seq based transcriptomes of a same sample give highly superimposable results. Various other declinations of the hardware part of this technology have emerged, such as to analyze more than just mRNA (*e.g.*, lcRNA, miRNA, …) in samples. Likewise, various transcriptomics computing algorithms have allowed to determine the genes differentially expressed (DEG) between two samples, the functional significance of DEGs by gene set enrichment analyses (GSEA) (12), as well as the inference of leukocyte cell composition of a tissue sample by deconvolution of its bulk transcriptome (*e.g.*, CIBERSORT) (13, 14), to quote a few. Since two decades, thousands of transcriptomics studies have been produced and were made freely available on public repositories (NCBI Gene Expression Omnibus (GEO), https://www.ncbi.nlm.nih.gov/gds, European Bioinformatics Institute’s Array Express https://www.ebi.ac.uk/arrayexpress/). Despite such significant improvements however, transcriptomics remained bulk and, by lacking the ultimate resolution of its single cells taken individually, was the mere arithmetic mean of its cell constituents.

Bulk RNA studies in lymphoma patients proved that TME signatures strongly impacted prognosis in (15, 16), besides genetic lesions. Deconvolution of publicly available gene expression profiles (GEP) datasets from 480 DLBCL patients treated with R-CHOP (standard therapy frontline) has allowed to draw maps of TME in 2015 (CIBERSORT), through characterization of cell composition from their GEP (this method has been applied to 18,000 human tumors and correlated leukocytes subsets to survival across various cancers) (14). However bulk transcriptomic analyses that rely on RNA extraction from pooled cell populations from tumor tissue cannot identify low abundant population or rare cell subtypes and could be not able to differentiate cells with similar expression patterns. The characterization of the complex relationship between tumor and bystander cells requires a correct transcriptional characterization at cellular level. Digital cytometry (CIBERSORTx) establishes molecular profiles and specific gene matrices from single cell or sorted cell transcriptomes, and uses them to “barcode” other samples with bulk RNA available to evaluate cellular abundance and GEP. This method has been applied to isolate 49 distinct transcriptional states across 13 signatures including macrophages, neutrophils, fibroblasts, and T cells. An atlas of ecosystems in DLBCL correlating to somatic mutations and distinct from tumor-based GEP classification has been built. Though more applicable to large datasets, this interesting approach remains less effective than genuine scRNA-Seq for understanding of the function and/or phenotype of individual cell types, even rare or unknown cellular subsets without signature matrix known.

## Implementation of scRNA-Seq: Technical Considerations

**Figure 1:**
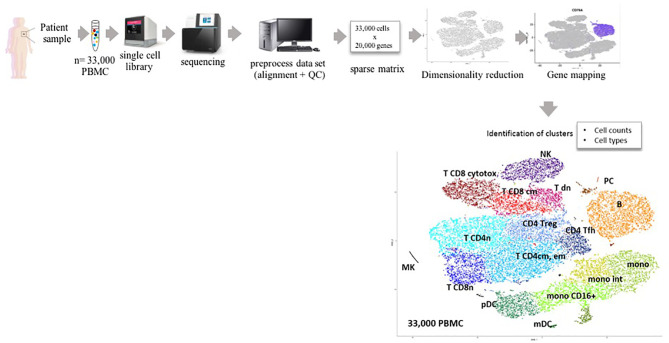
Sample flow chart for single cell RNAseq analysis. PBMC, peripheral blood mononuclear cells; PCA, principal component analysis; QC, quality control; t-SNE, t-distributed stochastic neighbor embedding. Legend for the t-SNE map:MK megakaryocytes, mono, monocytes; int, intermediate; m/pDC, myeloid/plasmacytic dendritic cells; NK, natural killer; PC, plasma cells, and for T cells: cytotox cytotoxic, dn, double negative; cm, central memory; em, effector memory; fh, follicular helper; n, naïve; reg, regulatory.

How we can tackle the obstacles discussed above with bulk RNA analyses? Among the huge development of single-cell technologies and computational advances to understand single cell transcriptomic profiling, the most commonly used technique remains scRNA-seq. This technology allows for precise determination of cell counts (with the limitation sometimes that across samples, in a heterogenous cell mixture, certain populations might be lost), cell types, and fine identification of the transcriptomic hallmarks of each cell cluster present in the initial sample. Whether based on microfluidics or on micro-wells, each single cell from a sample of thousand cells is processed individually. Each single cell from cell suspensions is successively lysed, all its mRNAs are captured through their 3’ end by an oligonucleotide carrying a cell-identifying tag, cut in smaller fragments (~100 nt), converted to cDNA, and amplified to produce a single cell library (capture can be done also by 5’ end, but this technique is not commonly used yet). All the single cell-derived and tagged libraries are pooled together to constitute the sample library. The library is then separately sequenced, each initial mRNA fragment becomes a “read,” yielding for the whole sample a file typically composed of 400 million reads, each corresponding to each initial mRNA fragment. In a further bioinformatics preprocessing of the sample, each read from this file is aligned *1)* on the sequence of the species transcriptome to identify its gene and *2)* to the cell-specific tag to identify its originating cell. This procedure is reiterated for all reads of the library such as to count how many reads are measured for each gene from each cell, yielding the so-called *(cell, gene)* matrix from the sample. Typically, a single cell RNA sequencing (scRNA-seq) matrix result comprises thousands of cells and about ten thousands of genes (since not all genes are detected and each cell does not express all the genes). Today, current scRNA-Seq technologies measure about <2,000 genes per cell. Further standard pre-processing of the data includes a normalization of all read counts and a quality control (QC) in which cells with too few genes, genes in too few cells, dead cells, and cell doublets are discarded from the dataset. A first step of data processing consists in clustering cells according to their gene expression profile, providing the most coherent and data-driven analysis of a mixed sample. To this aim, a principal component analysis is first performed to reduce the large dimensionality of all transcriptomes to their first principle components (PC). Once these fewer dimensions are selected upon user’s decision based on the wanted precision, clusters of cells with similar profiles are delineated under the same user’s criteria: low granularity makes less clusters of very different cells while more granularity means more clusters of more closely related cell types. Finally, the entire dataset is represented on bi-dimensional maps of cells, in which the above *n* first principle components are dimensionally reduced to two dimensions by sophisticated unsupervised algorithms such as t-distributed stochastic neighbor embedding (t-SNE). More recently, a superior method for both PCA and dimension reduction called uniform manifold approximation and projection (UMAP) has gained a large popularity for processing and visualization of scRNA-seq datasets (17). Within t-SNE or UMAP representations of those datasets, clusters are typically shown with colors and define cell subsets with high fidelity to the data: all cells alike are plotted next to each other while different cells form separate clusters with or without continuity to the former (**Figure 1**). Those steps are now performed by skilled bioinformatics users applying either R scripts from algorithms such as Seurat (18), or the proprietary Chromium™ (https://www.10xgenomics.com/) tools. Nevertheless, methods to infer cell types from scRNA-seq results are manifold, but still face challenges. Indeed, to identify a cell type, screening the dataset for cells expressing a specific marker gene is currently the most straightforward method. Data comprise many single cells in which many genes are often undetected for either biological (low expression level in the cell) or technical (read not sequenced or not captured) reasons.

## Applications of scRNA-Seq: In Search of the Cell of Origin and Subclonal Diversity

The first demonstration of a true insight of scRNA-Seq approaches has been a pioneer study in a germinal center (GC)-derived tumor, called follicular lymphoma (FL), which shed light on the ontogeny of FL cells (19). By comparing single-cell expression of a panel of 91 pre-selected genes in cancer *versus* normal B-cells, the authors demonstrated a clustering between FL, normal GC, memory, and antibody producing cells (plasma cells). They developed an original pseudo-time inference algorithm which suggested a developmental ordering of gene expression in the latter cells (from plasmablasts to mature plasma cell). Such modeling of maturation inferred from a panel of genes expression was then applied to phenotypically defined GC cells subsets (light zone LZ CXCR4^lo^CD83^hi^ and dark zone DZ CXCR4^hi^CD83^lo^), this algorithm indicated that 26% of the cells were misclassified, with an intermediate CXCR4/CD83 phenotype, proving that our vision of GC maturation was incorrect. Normal GC B cells spanned over a continuum of gene expression, not fully captured by the two states model, as they cyclically transitioned from LZ to DZ, with about one third of cells being in an intermediate state sharing LZ/DZ markers. Furthermore, correlations (and anti-correlations) between discrete clusters of genes allowed to demonstrate a synchronized gene expression program defining identity of normal GC cells. Avoiding averaging effects of mRNA bulk analysis, scRNA-Seq study of five FL patients at diagnosis clearly showed heterogeneity of gene expression patterns within and between patients’ samples, not seen in normal GC B cells. By comparing the 91 genes expression profiles, FL cells clustered separately from GC or memory cells, and with a major desynchronization of the GC-specific program, suggesting that despite sharing a common ancestral signature FL cells are genetically different from their putative cell of origin. Sample origin was a major source of genetic heterogeneity across the 5 FL patients, but intra-patient heterogeneity was not linked to subclonal genomic diversity, since *IGH* subclones tracking found that a given subclone could be found in different states (at least in the 91-gene panel). A second study (20) applied scRNA profiling to dissect the heterogeneity of GC tumors cell-of-origin (sc-COO). Given inter-donor consistency, in terms of gene signature associated to specific subsets of GC cells, they identified multiple functionally linked subpopulations (with some cells showing intermediate level of GC differentiation process between DZ/LZ), as well as the precursors of both memory B cells and antibody secreting cells (based on expression of *CCR6* and *PRDM1*). Moreover, a gene classifier (selecting the 50 up- and down-regulated genes in the sc signatures associated with these GC subpopulations) was built and applied on bulk RNA-Seq expression profiles from two published DLBCL panels (481 fro NCI and 230 from BCA). Non-GC cases were scattered across the sc-RNA Seq classes (but mostly in late GC stages) while the majority of GC cases were related with normal GC B cells in the LZ (like early of intermediate stages). Interestingly, 12% of DLBCL cases in both datasets displayed DZ gene signatures despite distributing across GC/non-GC/unclassified tumors. Together with an enrichment in double-hit (MYC/BCL2) cases, these results suggest a different ontogeny for this high-risk subset of DLBCL. Progression-free survival after R-CHOP was found to be dependent across the 5 prognostic categories isolated from the baseline 13 sc-*coo* signatures, proving that the latter identified clinically relevant subgroups of DLBCL patients.

To explain how these states are defined, cell-intrinsic genetic events and/or interactions with the microenvironment are possible. Mutations of epigenetic regulating genes are seen in 100% of FL cases (21–23) because they are key in the processes of initiation, apoptosis resistance, and progression of the disease, such as loss-of-function *KMT2D* (23) or *CREBBP* mutations (24, 25), or gain-of-function *EZH2* mutations (26). But besides those genes, genomic landscape of FL dramatically influences the nature of the ecosystem, as recently demonstrated with *CREBBP* (25), cathepsin S (*CTSS)* (27), *EZH2* (28), and *TNFRSF14/HVEM* (29) mutations. On the one hand, activation of *EZH2* gene attenuates T follicular helper (T_FH_) cells’ help for proliferation (indispensable to normal GC B cells differentiation) in FL cells, and drives slow expansion of centrocytes the characteristic niche of the tumor, embedded within the Follicular Dendritic Cells meshwork (30). On the other hand, loss of HVEM leads to FL proliferation by inducing tumor supportive ME with increased stroma activation and T_FH_ cells recruitment (31, 32). Lastly, *CREBBP* loss-of-function mutations (genocopied by *TET2* loss-of-function mutation) contribute to immune evasion *via* a decrease of class II trans-activator (CIITA)-dependent MHC class II expression (of both transcript and protein), leading in the tumor bed to a decrease of infiltration of both CD4+ helper and CD8+ memory cytotoxic T cells (25). Very seemingly, *CTSS* gain-of-functions mutations or amplifications allow for an enhanced MHC class II-restricted antigen presentation to CD4+ T cells, with better prognosis for patients.

But detecting genetic variants, such as mutations and copy number alterations, in scRNA-Seq reads is not an easy task. Tools have now been developed to study subclonal complexity of a tumor. Mutations can now be identified at the single cell level, to cluster tumor from normal cells, derive mutation-specific gene signatures, identify cell surface markers, and build phylogenetic trees of subclonal driver genes evolution, as shown in acute myeloid leukemia (31) (this has never been done in lymphomas). This latter study acquired transcriptional and mutational data in 16 AML and 5 normal bone marrows to profile AML tumor ecosystems changes with therapy, and demonstrate for the first time that differentiated AML had immune-modulating properties against T cells. There is no such study to date in lymphomas, the scRNA-Seq profiling is being extensively used to build an atlas of tumor *versus* normal immune cells within specific micro-environments.

Still, indirect evaluation of oncogenes mutational status can be inferred from scRNA-seq approaches. In another study in six FL patients and five control specimens (32), authors managed to assign cells to eight different lineages, or immune subsets, in concordance with a 13-parameter FACS analysis for B, T, and NK cells but not monocytes (it is likely that development of CITE-Seq will tackle this kind of discordance). As expected from the study by Milpied et al. (19), evolutionary paths (different subclonal somatic mutations) followed by pre-malignant B cells to FL cells could be inferred from transcriptomic heterogeneity (normal B cells on the other hand clustered together). Differential expression analysis revealed transcription of genes specific to the tumor *versus* the normal B cells within a patient’s sample. But most interestingly, samples with CREBBP mutation indeed had lower expression of *HLA-DR* gene than wild-type samples, but also lower expression by two separated clusters of quiescent subpopulations within a single tumor, suggesting scRNA-Seq captured transcriptomic consequences of genomic alterations inter- and intra-patients. In this study, coding somatic mutations deeply modified the expression of sets of genes, structuring the tumor into various subclones based on their genetic disparities (4–5 per sample), the sizes of which were similar regarding on the method used to quantify them (scRNA-Seq and exome-Seq). Therefore, gene expression heterogeneity, at least in part, was also attributable to subclonal genomic heterogeneity, even if other drivers of phenotypic diversity are stronger as proposed by the preceding study. Lastly, authors investigated T cells subsets and gene expression in double immune checkpoint expressing cells, confirming expression of genes inhibiting T-cell activation in CD4+ memory T cells co-expressing *TNFRSF18* and *TNFRSF4*. Overall, droplet-based scRNA-Seq with 10,000 cells only demonstrated its power to analyze tumor heterogeneity and infiltrating T cells phenotype.

## Applications of scRNA-Seq: Deciphering Functional Diversity Within Ecosystems

The tumor microenvironment (TME) is constituted by heterogeneous cellular populations including tumor cells and the surrounding non-malignant cells, such as numerous and distinct immune cells and stromal cells. Beyond the heterogeneity of the tumor cells (see above COO), the diversity and plasticity of the microenvironment also contributes to the intra-tumor heterogeneity (7, 33, 34). Strong evidences show that diverse immune subsets and their interactions within the tumor microenvironment are critical to diverse aspects of tumor biology, treatment response, and prognosis (35, 36). To avoid the need for manual annotation of cell types to existing data after unsupervised clustering, a probabilistic model [CellAssign (37)] has been developed, able to statistically frame the analyses of TME across samples and cancers by assigning cells to both known and *de novo* cell types in scRNA-seq data.

The TME is always a mix of lymphoma and normal cells [with Hodgkin’s disease (HD) even as a paradigm of TME cells largely overwhelming the number of Reed-Sternberg cells] (38). Thus, a perfect isolation of the malignant cell population from a surgical biopsy is a significant challenge especially when tumor cells are low abundant. TME composition could be extremely variable according to the invaded sites (blood, bone marrow, or lymphoid secondary organs). It could be composed of extra-cellular matrix and stromal cells (shaping the architecture of the lymph node and T-B cells contacts), innate (myeloid and lymphoid) and adaptive immune cells, and vascular (blood and lymphatics for homing/egress from niches) cells (39, 40).

To date, the most detailed functional and spatial profiling of TME cells at the single cell resolution has been published in HD (41). Authors performed scRNA-Seq characterization of immune cells and assessed their spatial sub-localization out of 22 HD patients and five reactive lymph nodes. Transcriptome data from >100,000 cells and 1,200 genes in median identified 22 clusters, all being assignable to a cell type based on the published transcriptomes of sorted immune cells. No tumor specific cluster was found. But three regulatory T cells (Treg) clusters dominated the TME of HD. The cluster in HD cases with the highest proportion of immune cells was also enriched in LAG3 and CTLA transcripts, as opposed to controls where B cells and CD8+ T cells were enriched. Non-Treg CD4+ clusters also enriched in HD included Th2 and Th17 subsets. Treg CD4+ subsets in HD cases expressed GITR, CD25, not FOXP3 (and thus endowed with a type 1 Tr1 phenotype). Inhibitory receptor-mediated immune tolerance of HD cells is further reinforced by the co-localization of LAG3+ Treg near MHC class II-negative tumor cells by multicolor immunohistochemistry. Since FOXP3+ Treg were significantly reduced in the later samples (increased only in CMH class-II positive tumors), LAG3+ Treg are thus considered a disease-specific subset. In an independent series of 166 patients treated with standard ABVD regimen, IHC confirmed that expression of LAG3+ T cells correlated with the loss of CMH-class II by tumor cells, with no impact on prognosis.

Besides HD, ecosystem of cutaneous T-cell lymphomas (CTCL) has been evaluated in a study applying scRNA-Seq focusing on 14,000 CD3+ T lymphocytes (and a median of 1,200 genes) from four healthy skin donors and five advanced stage patients. The results revealed large inter-patient heterogeneity and no overlap with normal skin samples (42). Twenty-six clusters were identified and cell types annotated with normal dermis signatures. Greater heterogeneity was found at the level of lymphocytes, macrophages, keratinocytes, and fibroblasts. A gene expression signature identifies highly proliferative T cells, among which 17 genes were shared by all five patients’ samples, including two markers assessable by IHC to diagnose aggressive CTCL. Over-expression of TIGIT, LAG3 and TIM3 by CD8+ and CD4+ T cells (exhaustion signatures) indicated strong rationale for immunotherapies in this disease. Presence of TIGIT+ Treg correlated with lack of granzyme B and perforin in infiltrating CD8+ T cells. Such differences in malignant and reactive T lymphocytes in CTCL have been also reported in circulating form of a CTCL subtype (mycosis fungoides), called Sézary syndrome. Another group investigated this latter condition using scRNA-Seq and unraveled the importance for disease evolution of transitioning from *FOXP3+* malignant T cells to *GATA3+* or *IKZF2+* cells. This transcriptional heterogeneity could be used to inform on prognosis, with FOXP3 and another set of 19 genes being involved in early progression in CTCL cases (43).

Beyond investigating heterogeneity, scRNA-seq has been also used to study putative cell-to-cell communications, inferred from the correlation of expression levels of paired ligand and receptor of individual cells (without knowing spatial proximity) (44–46). These original approaches have been extended thanks to an algorithm called NicheNet (47). One single example of the application of this computational approach in nine lymphoma patients has been published (48). This analysis suggested that cancer B cells could receive signals from all four major subsets of T cells (especially T follicular helper subset as the major source for IL-4, a putative resistance mechanism against ibrutinib). These dynamic stromal niches, as already reported in solid cancers (49), fully support outgrowth of lymphoma cells.

Other studies using scRNA-seq have also focused on depicting differentiation single-cell states, pro and antitumor function of immune cell and their distributions in cancer (50). Furthermore, the ability to define the TCR sequence at a single-cell level enabled to analyze the association between therapeutic response and activation states of specific T cell clusters (51, 52). Then, the development of single-cell transcriptomic technology enables to analyze the heterogeneity of immune subsets within the TME and emerges as powerful tools to screen immune-related signatures and identify potential biomarker which may as prognostic factors or therapeutic targets (53–55). International efforts attempting to set up a cancer human atlas at the single-cell resolution have been fruitful (3, 56), delineating immune contexture and activation state, for prognosis prediction and immunotherapy guidance in solid cancers. But data in lymphomas are still quite scarce (57).

## scRNA-Seq and Clinical Research: Monitoring Response to Therapy and Understand Resistance/Toxicities

Most personalized therapies do not take into account heterogeneity of nodal lymphomas. A previously discussed paper elegantly investigated both malignant and non-malignant lymphocytes in 12 donors (nine with lymphoma and three reactive lymph nodes) (48). Authors have found coexistence of up to 4 transcriptionally distinct subpopulations of lymphoma cells, responding differently to treatments *in vitro* and *in vivo* in an example assessed at the time of relapse. This scRNA-Seq molecular profiling of transcriptomic signatures of resistant subclones will undoubtedly help tailor better therapies in each patient, and how resistance subclones evolve over time (clonal competition).

In relapsed/refractory DLBCL patients receiving CD19 chimeric antigen receptor T-cells (CAR T-cells), scRNA-Seq has been used to investigates biomarkers of early progression, but also of toxicities [cytokine release syndrome (CRS), immune cell activation neurologic syndrome (ICANS)]. Previous studies had highlighted the implications of T-cells subsets in the success of therapy. By doing scRNA-Seq analysis of CAR products in 24 patients, authors of a very recently published paper identified that exhausted T cell phenotype was more abundant in patients not entering complete response (CR) (58). But they also identified a very small subset of IL-1β+ and IL-8+ myeloid cells (<300) associated with more severe ICANS. These results, together with the explanation of scRNA-Seq curated data showing expression of CD19 by mural cells maintain brain-blood barrier integrity (therefore targeted by CARs) (59), elegantly demonstrated that efficacy and toxicity of immunotherapies can both be optimized.

Though not yet with an obvious application in the therapy of lymphoma, scRNA-Seq studies have been extensively used to predict immunotherapy responses (especially immune checkpoint inhibitors, or ICIs) in various solid cancers, based on T-cell infiltrating lymphocytes (TILs)’s characteristics in the TME, that target neo-antigens [review in (60)]. TILs in different cancers have proved to share common signatures, but also possess specific characteristics in line with the organized TME they reside in. After ICIs exposure, two studies published in 2018 (61, 62) have defined melanoma-specific and TILs-specific transcriptome signatures associated with outcomes. By pairing scRNA-Seq with TCR sequencing, two other groups showed that ICI induced expansion of T cells with different clonotypes (meaning recruitment of peripheral CD8+ T cells inside the tumor bed), rather than boosted the pre-existing TILs (63, 64). Paucity of myeloid cells subsets were also correlated to outcomes in another study (41). With the development of more and more ICIs strategies, in many cancers, and with the help of single cell-based technologies, this a revolution surging in the field of immunology, not only onco-immunology (65, 66).

## Upcoming Improvement to Unravel TME From scRNA-Seq Approaches

A major improvement has recently been brought by implementing scRNA-seq with use of DNA oligonucleotide-tagged antibodies (ADT), such as to integrate cell surface proteins together with transcriptome measurements. The resulting technique, called cellular indexing of transcriptomes and epitopes by sequencing (CITE-seq), analyses ADT-labeled cells by scRNA-seq to provide simultaneously both immuno-profiling and transcriptomics of the same single cells (67). ADT-labeling enhances the identification of each cellular population present in the samples, and avoid further misinterpretation of results. Data can be processed with dedicated software, such as Single-Cell_Signature_Explorer and single cell virtual cytometer, the first published method for qualitative and scoring of single gene or gene-set based signatures (68). This method could thus provide informations about: cellular heterogeneity and evolution (our data, *in press*), clues of cell maturation through pseudo-temporal trajectory analysis (74) or cellular dynamics by RNA velocity determination (70), or definition of GC differentiation state based on CXCR4 and CD86 proteins expression levels, thus confirming sc transcriptomics data (20).

By its compatibility with various declinations of the scRNA-seq technology, the results of such multimodal analyses allow to reach an unapproached level of multi-omics characterization. On the other hand, detecting genetic variants, such as mutations and copy number alterations, in scRNA-Seq reads is not an easy task. Tools have now been developed to study subclonal complexity of a tumor. Mutations can now be identified at the single cell level, to cluster tumor from normal cells, derive mutation-specific gene signatures, identify cell surface markers, and build phylogenetic trees of subclonal driver genes evolution (31). More recently multi-omics single cell strategies are focused to study genetic, epigenetic, phenotypic, and transcriptomic profiles within the same cell. As examples, SCI-seq (single cell combination marker) provides cell copy number variation (71), scATAC-seq could identifies specific chromatin motifs (72), RAGE-Seq (repertoire and gene expression by sequencing) identifies B or T cell repertoire (73), scNGS could provide information about somatic mutations (1). All these approaches combined to new computational software should give huge knowledge of cellular heterogeneity, evolution, dynamics of lymphoma cells within their TME. Multi-omics (three techniques) evaluation of seven patients with chronic lymphocytic leukemia (CLL) exposed to ibrutinib has unraveled a consistent regulatory program of treatment-induced changes, but at a pace that varied among patients. First events were signs of NF-κB inhibition, followed by reduce activity of lineage transcription factors, resulting in erosion of CLL cells’ identity and after a few months, acquisition of a dormant, quiescence-like gene signature (72). This important study is the first to suggest that multi-omics approaches, combined with multi-timepoints analyses, are able to predict the molecular response to a kinase inhibitor in lymphoma patients.

## Conclusion

To advance cancer research and resolve heterogeneity, we need integrated single cell multi-omics platforms to study cell-by-cell (tumor, immune, stroma) transcriptomes, proteomes, methylomes, cell surface proteins, localization within the tumor, and even future evolution through (pseudo-time analysis), across various patients’ datasets. The information of the precise localization of normal immune *versus* tumor cells will be resolved in part through spatial transcriptomics. Thanks to newer bioinformatics tools, we need to understand the functional significance of the different clusters, better visualize them, and aggregate our data, from labs to labs all together, in a global effort of a multi-modal integration across generated datasets of tumors worldwide. These efforts will be paid back by enhanced risk stratification, disease monitoring, and personalized therapy in lymphomas, like a few studies in multiple myeloma and acute myeloid leukemia have already demonstrated.

## Author Contributions

LY, AQ-M, CL, MT, FP, and J-JF wrote the manuscript and analyzed the data. All authors contributed to the article and approved the submitted version.

## Conflict of Interest

The authors declare that the research was conducted in the absence of any commercial or financial relationships that could be construed as a potential conflict of interest.
